# Structural relation matching: an algorithm to identify structural patterns into RNAs and their interactions

**DOI:** 10.1515/jib-2020-0039

**Published:** 2021-05-31

**Authors:** Michela Quadrini

**Affiliations:** University of Camerino, School of Science and Technology, via Madonna delle Carceri, Camerino, Italy

**Keywords:** core, loops, relation matrix, relations, shape, structural pattern

## Abstract

RNA molecules play crucial roles in various biological processes. Their three-dimensional configurations determine the functions and, in turn, influences the interaction with other molecules. RNAs and their interaction structures, the so-called RNA–RNA interactions, can be abstracted in terms of secondary structures, i.e., a list of the nucleotide bases paired by hydrogen bonding within its nucleotide sequence. Each secondary structure, in turn, can be abstracted into cores and shadows. Both are determined by collapsing nucleotides and arcs properly. We formalize all of these abstractions as arc diagrams, whose arcs determine loops. A secondary structure, represented by an arc diagram, is pseudoknot-free if its arc diagram does not present any crossing among arcs otherwise, it is said pseudoknotted. In this study, we face the problem of identifying a given structural pattern into secondary structures or the associated cores or shadow of both RNAs and RNA–RNA interactions, characterized by arbitrary pseudoknots. These abstractions are mapped into a matrix, whose elements represent the relations among loops. Therefore, we face the problem of taking advantage of matrices and submatrices. The algorithms, implemented in Python, work in polynomial time. We test our approach on a set of 16S ribosomal RNAs with inhibitors of *Thermus thermophilus*, and we quantify the structural effect of the inhibitors.

## 1 Introduction

**Figure 1:**
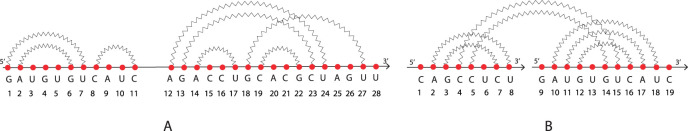
On the right (A), the diagram of an RNA secondary structure, on the left (B) an RNA–RNA interaction structure.

**Figure 2:**
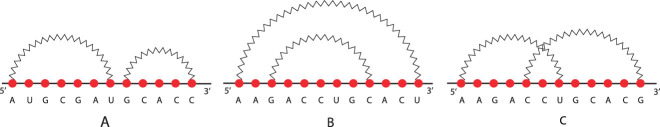
Relations between two loops: (A) concatenation, (B) nesting and (C) crossing of two loops.

Ribonucleic acid (RNA) is a linear polymer with a preferred 5–3′ direction, made of four different types of nucleotides, known as Adenine (A), Guanine (G), Cytosine (C), and Uracil (U). Each nucleotide is linked to the next one by a phosphodiester bond, referred to as a strong bond. It can at most interact with another non-contiguous one, establishing a hydrogen bond, referred to as a weak bond, and forming mainly Watson–Crick (G–C and A–U) and wobble (G–U) base pairs. Such a process, known as the folding process, induces complex three-dimensional configurations (or shapes). Each of them is strictly related to the molecular functions. RNA molecules play numerous roles in cellular processes. Usually, they do not act in isolation, but they express their biological roles by interacting with other molecules [1], including other RNAs that determine the so-called RNA–RNA interactions (RRIs). Understanding the link between shape and function has been considered a challenge in biology. Disregarding the molecular spatial configuration and reducing nucleotides to dots, RNAs can be abstracted in terms of secondary structures that consist of the nucleotide bases paired by hydrogen bonding within its sequence. This abstraction represents an intermediate level between the nucleotides sequence and its shape, and it is both tractable from a computational point of view and relevant from a biological perspective. For example, under the action of inhibitors, many 16S ribosomal RNAs alter their shape by preserving the nucleotides sequence [2], and the secondary structure can capture such changes. Moreover, functional RNA families, such as tRNA, rRNA, and RNAse P, exhibit a highly conserved secondary structure but little sequence similarity [3]. Searching for sequence motifs does not work effectively with RNA, while it has been a powerful tool for DNA and protein analysis [4]. Therefore, the ability to compare and classify RNA secondary structures equipped to identify common substructures is of great interest. A way for schematically representing a secondary structure is the arc diagram, constituted by vertices that formalize the nucleotides on a straight line (backbone) and semicircular zigzag arcs in the upper half-plane that depict the weak bonds. In the following, we will call this the diagram of the structure. A secondary structure represented by a diagram is pseudoknot-free if it does not present crossings among the weak bond zigzag arcs; otherwise, it is called pseudoknotted. On the right of Figure 1A, the RNA structure is pseudoknot-free, and on the left, we have a pseudoknotted motif, which makes the whole structure pseudoknotted. An example of RNA–RNA interaction structure in terms of arc diagram is shown in Figure 1B. Each arc determines a loop. Therefore, every RNA secondary structure is composed of loops. Given two of them, we have only three situations: a loop is followed by the other one as illustrated in Figure 2A, a loop is inside another one as shown in Figure 2B, and a loop crosses with the another as illustrated in Figure 2C. We refer to such relations as concatenation, nesting, and crossing, respectively. Some approaches have been exploited to study the link between the structure and its biological functions. Maestri and Merelli studied the relationships between RNA structure and functions by using process calculi [5]. Quadrini et al. introduced algebraic languages for representing and comparing RNA secondary structures with arbitrary pseudoknots [6], [7], while Andersen et al. exploited a combinatorial approach [8]. On the other hand, Giegerich et al. introduced the concept of shape [9], while Bon et al. proposed a classification of RNA secondary structures based on a topological invariant, the genus [10]. Reidys et al. developed several algorithms for predicting pseudoknots by exploiting the concept of shadow [11], [12]. Moreover, several approaches have been proposed for searching common patterns. Algorithms based on tree data structures find the largest approximately common substructures and patterns in [13] and [14], respectively. Affix trees allow us to exact and approximate pattern matching [15]. Arslan et al. proposed a substructure search algorithm based on a binary search on a suffix array to find the largest common substructure of given RNA structures [16]. Backofen and Siebert have developed a dynamic programming approach for computing common exact sequential and structural patterns between two RNAs without pseudoknots [17]. Several proposed approaches have been based on arc-annotated sequences, also called contact maps, including the longest arc-annotated subsequence problem, the arc preserving subsequence problem, the maximum arc-preserving common subsequence problem, and the edit-distance for arc-annotated sequence problem [18]. Blin et al. introduced an approach, called maximum arc-preserving common subsequence problem to compare arc-annotated sequences in [19], and Evans proposed an algorithm to find common structures excluding some classes of pseudoknots [20]. Recently, Quadrini et al. have faced the problems of identifying substructures considering both the primary and secondary structure only for RNA pseudoknot-free structure in [21].

**Figure 3:**
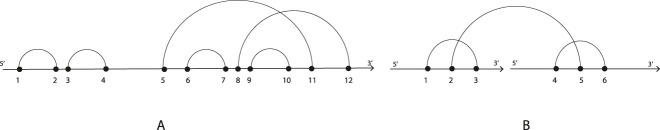
The core of the structures shown in Figure 1.

In this paper, we face the problem of identifying a given structural pattern into the secondary structure of RNAs and or RNA–RNA interactions. We propose a method that works on patterns and structures characterized by arbitrary pseudoknots. Following the approaches proposed in [22], [23], we introduce the concept of the core. The core of a secondary structure is determined by deleting unpaired nucleotides and by collapsing arcs, appropriately. For example, the core of the structures in Figure 1 is shown in Figure 3.

**Figure 4:**
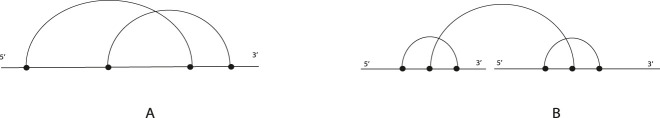
The shadow of the structure shown in Figure 1.

In the literature, the concept of collapsing has already been introduced to reduce the complexity and classify the RNAs in equivalent classes based on topological concepts. From each diagram, it is possible to associate a shadow by removing all the non-crossing arcs and all the unpaired vertices, and then collapsing parallel zigzag arcs into an arc [24]. For example, the shadow of the structures in Figure 1 is illustrated in Figure 4. The shadow is again a diagram that is unique for the considered molecule, like to core. Based on our previous results [6], [25], we define three operators able to formalize the concatenation, nesting, and crossing between two loops. Such operators are necessary and sufficient to describe any arc diagram in terms of relations among loops. Such description allows us to uniquely associate a matrix, called relation matrix, whose elements represent the relation between the two corresponding loops for each abstraction (secondary structure, core, or shadows) of RNA and RNA–RNA interactions structures. Therefore, identifying a given structural pattern into an RNA structure corresponds to search a submatrix within a matrix. To reach the aim, we have defined four algorithms: loop determination, core determination, determination of the relation matrix, and structural relation matching. Each of them is implemented in Python and works in polynomial time. The first three algorithms take as input a secondary structure of an RNA or RNA–RNA interaction encoded as a Bpseq notation and return the list of loops of the secondary structure, of the core and the shadow, respectively. A Bpseq notation contains information about base pairs, stored in three columns: the first one represents the sequence position, the second contains information about the kind of bases (i.e., Adenine, Guanine, Cytosine, or Uracil), the third encodes the pairing base if the considered nucleotide is paired, or zero, otherwise. The other algorithm considers the output of the previous one, and it returns the relation matrix. The last one searches the relation matrix associated with the pattern into the relation matrix of the structure. The approach has been tested on structures of 16S ribosomal RNAs from the RNA Strand database [26]. In particular, we have selected 16S ribosomal RNAs with inhibitors of *Thermus thermophilus* to quantify the structural effect of the inhibitors.

The paper is organized as follows. In Section 2, we formally introduce the arc diagram representation within the concepts of core and shadows. Moreover, we describe the relations among loops and we define the relation matrix. In Section 3, we formally face the problem of searching a structural pattern. In Section 4, we present an application of our methodology on a set of 16S ribosomal RNAs. The paper ends with some conclusions and future perspective.

## 2 RNA abstractions and representation

RNA secondary structure can be represented by a diagram. Formally,

**Definition 1**

**(Diagram)**. A diagram is a labeled graph over the ordered set of vertices [*ℓ*] = {1, …, *ℓ*}, in which each vertex has degree ≤3, and the edges are all the segments [*i*, *i* + 1] for *i* = 1, …, *ℓ* − 1 and some semi-circular arcs (*i*, *j*) in the upper half-plane, with 1 ≤ *i* < *j* ≤ *ℓ*.

The diagram is denoted by D=(ω,B) , where *ω* is the string that corresponds to the sequence of labels over the ordered set [*ℓ*] and *B* is the set of all arcs (*i*, *j*). In the literature, this notation represents arc annotated sequences. To not introduce many symbols, we denote the arc diagram by the pair (*ω*, *B*) since only arc diagrams are considered in this work. Moreover, a diagram corresponds to an arc annotated sequence, whose nodes have a degree less than or equal to 3. As an example, the structure illustrated in Figure 1A is formalized by (GAUGUGUCAUCAGACCUGCACGCUAGUU, {(1, 7), (2, 6), (8, 11), (12, 24), (13, 23), (14, 17), (18, 27), (19, 22)}), while the secondary structure of RNA–RNA interaction is identified by (CAGCCUCUGAUGUGUCAUC, {(2, 8), (3, 7), (4, 15), (5, 14), (10, 18), (11, 17), (12, 16)}). We associate the core to each diagram by deleting the unpaired nucleotides and by collapsing parallel arcs. A nucleotide is unpaired if it has not arc incident upon it, while two arcs, identified by the pairs (*i*_1_, *j*_1_) and (*i*_2_, *j*_2_) are parallel if and only if *i*_1_ = *i*_2_ − 1 and *j*_2_ = *j*_1_ + 1 or vice-versa. As an example, the core of the structure in Figure 1A is identified by (*ϵ*, {(1, 2), (2, 4), (5, 11), (6, 7), (8, 12), (9, 10)}). Note that since the core is obtained by deleting nucleotides, the string *ω* of the core is empty, *ϵ*. Similarly, the shadow of the structure in Figure 1A is (*ϵ*, {(1, 3), (2, 4)}). To obtain the secondary structure, core, or the shadow in terms of loops, we define three procedures: loop determination, core determination, and shadow determination. These procedures take as input a secondary structure of an RNA or RNA–RNA interaction encoded as a Bpseq notation, and return a set of loops of arc diagram related to the secondary structure (Algorithm 1), to the core and shadow, (Algorithm 2) respectively. As described in the Introduction, each arc diagram is composed of loops. Given two loops, *L*_*s*_ and *L*_*t*_, there are only three possible relationships between them. We say *L*_*s*_ is concatenated to *L*_*t*_ (*L*_*s*_ ⊙ *L*_*t*_) if the vertices of the relative pairs (*i*_*s*_, *j*_*s*_), (*i*_*t*_, *j*_*t*_) satisfy the following relation *i*_*s*_ < *j*_*s*_ < *i*_*t*_ < *j*_*t*_. We say *L*_*s*_ is nested into *L*_*t*_ (*L*_*s*_ ⋒ *L*_*t*_) if *i*_*t*_ < *i*_*s*_ < *j*_*s*_ < *j*_*t*_ and we say *L*_*s*_ crosses with *L*_*t*_ (*L*_*s*_ 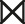 *L*_*t*_) if *i*_*s*_ < *i*_*t*_ < *j*_*s*_ < *j*_*t*_. Without loss of generality, we enumerate loops of the structure starting from the loop whose last nucleotide is the most left. In other words, the first loop *L*_1_ is formed by the pair (*i*_1_, *j*_1_) such that *j*_1_ is the last paired nucleotide of the structure considering the 5–3′ direction. As a consequence, given two loops *L*_*s*_ and *L*_*t*_ respectively formed by (*i*_*s*_, *j*_*s*_) and (*i*_*t*_, *j*_*t*_), if *s* < *t* then by definition *j*_*s*_ > *j*_*t*_. As an example, we can consider the structure illustrated in Figure 5 that consists of nine loops, *L*_1_ = (2, 6), *L*_2_ = (4, 11), *L*_3_ = (8, 13), *L*_4_ = (10, 18), *L*_5_ = (16, 19), *L*_6_ = (15, 20), *L*_7_ = (9, 24), *L*_8_ = (22, 27), *L*_9_ = (21, 28), and 36 relations among them, i.e., *L*_1_ 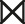 *L*_2_, *L*_1_ ⊙ *L*_3_, *L*_1_ ⊙ *L*_4_, *L*_1_ ⊙ *L*_5_, *L*_1_ ⊙ *L*_6_, *L*_1_ ⊙ *L*_7_, *L*_1_ ⊙ *L*_8_, *L*_1_ ⊙ *L*_9_, *L*_2_ 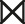 *L*_3_, *L*_2_ 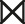 *L*_4_, *L*_2_ 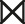 *L*_5_, *L*_2_ ⊙ *L*_6_, *L*_2_ ⊙ *L*_7_, *L*_2_ ⊙ *L*_8_, *L*_2_ ⊙ *L*_9_, *L*_3_ 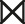 *L*_4_, *L*_3_ 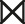 *L*_5_, *L*_3_ ⊙ *L*_6_, *L*_3_ ⊙ *L*_7_, *L*_3_ ⊙ *L*_8_, *L*_3_ ⊙ *L*_9_, *L*_4_ ⋒ *L*_5_, *L*_4_ ⋒ *L*_6_, *L*_4_ ⋒ *L*_7_, *L*_4_ 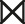 *L*_8_, *L*_4_ 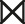 *L*_9_, *L*_5_ 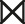 *L*_6_, *L*_5_ 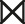 *L*_7_, *L*_5_ ⊙ *L*_8_, *L*_5_ ⊙ *L*_9_, *L*_6_ ⋒ *L*_7_, *L*_6_ ⊙ *L*_8_, *L*_6_ ⊙ *L*_9_, *L*_7_ ⊙ *L*_8_, *L*_7_ ⊙ *L*_9_, *L*_8_ ⋒ *L*_9_.

**Figure 5:**
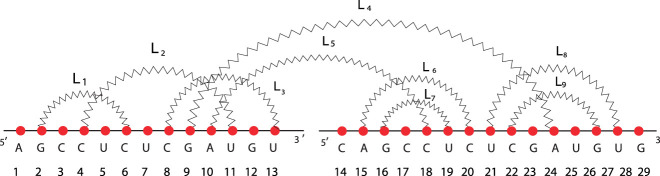
Example of an RNA secondary structure.

Taking advantage of such an enumeration, we impose an order on the loops. Each nucleotide can interact at most with another one. As a consequence, each nucleotide can be involved at most in a pair. This means that the choice of a loop is unique. Moreover, the three relations, concatenation, nesting, and crossing, are necessary and sufficient to describe any RNA secondary structure with arbitrary pseudoknots. In fact, given two loops, *L*_*s*_ and *L*_*t*_ with *s* < *t*, it is equivalent to consider the two pairs of natural number (*i*_*s*_, *j*_*s*_) and (*i*_*t*_, *j*_*t*_) such that *i*_*s*_ < *j*_*s*_, *i*_*t*_ < *j*_*t*_ and *j*_*s*_ < *j*_*t*_. It follows that *j*_*s*_ is the greatest number. From the theory of combinations, we have 6 different order relations over *i*_*s*_, *j*_*s*_, and *i*_*t*_, which became 3 considering the constraints *i*_*s*_ < *j*_*s*_ and *i*_*t*_ < *j*_*t*_. For each structure, we can uniquely associate a relation matrix. Each element *a*_*ij*_ of the matrix is the relation between the loops *L*_*i*_ and *L*_*j*_. The relation matrix of the structure in Figure 5 is given in Table 1. By appropriately exchanging some rows and columns, we obtain the following matrix that allows distinguishing the relations between the two molecules and the relations due to the interaction (Table 2). In this way, for each RNA–RNA interaction structure, we can identify structural patterns of the two single molecules or interactions between them. The ability to distinguish if a set of loop relations characterize the interactions between molecules or a single molecule is useful for studying how the molecules interact with each other us allowing to study how such interactions change according to the environment.

**Table 1:** The relation matrix of the RNA secondary structure shown in Figure 5.

	*L*_1_	*L*_2_	*L*_3_	*L*_4_	*L*_5_	*L*_6_	*L*_7_	*L*_8_	*L*_9_
*L*_1_	–	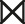	⊙	⊙	⊙	⊙	⊙	⊙	⊙
*L*_2_		–	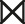	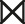	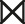	⊙	⊙	⊙	⊙
*L*_3_			–	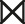	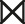	⋒	⋒	⋒	⋒
*L*_4_				–	⋒	⋒	⋒	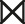	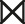
*L*_5_					–	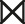	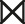	⊙	⊙
*L*_6_						–	⋒	⊙	⊙
*L*_7_							–	⊙	⊙
*L*_8_								–	⋒
*L*_9_									–

**Table 2:** The relation matrix of the secondary structure of RNA–RNA interaction shown in Figure 5.

	*L*_1_	*L*_2_	*L*_3_	*L*_4_	*L*_5_	*L*_6_	*L*_7_	*L*_8_	*L*_9_
*L*_1_	–	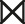	⊙	⊙	⊙	⊙	⊙	⊙	⊙
*L*_2_		–	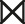	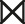	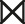	⊙	⊙	⊙	⊙
*L*_4_				–	⋒	⋒	⋒	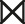	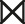
*L*_5_					–	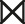	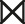	⊙	⊙
*L*_3_			–	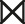	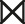	⋒	⋒	⋒	⋒
*L*_6_						–	⋒	⊙	⊙
*L*_7_							–	⊙	⊙
*L*_8_								–	⋒
*L*_9_									–

## 3 Structural matching

**Figure 6:**
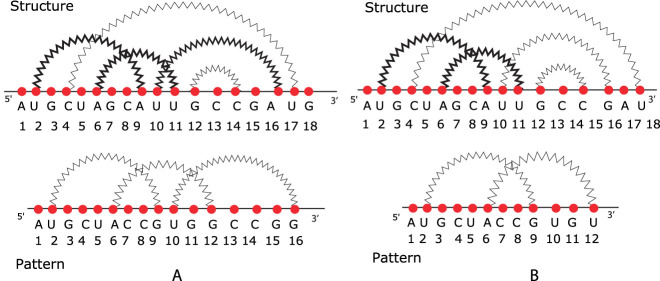
(A) A graphical example of APS problem and (B) an illustration of the problem with our restriction.

We face the problem of searching a given structural pattern into a secondary structure or its abstraction, i.e., core and shape, of RNAs and RNA–RNA interactions with arbitrary pseudoknots. Formally, we address the problem of the arc-preserving subsequence (APS) problem with a particular restriction. Let D=(ω,B) , and $D^{\prime }=\left(\omega^{\prime },B^{\prime }\right)$ be two arc-annotated sequences such that *n* = |*ω*| and *m* = |*ω*′| with *n* ≥ *m*, the APS problem asks whether D can be exactly obtained from $D^{\prime }$ by deleting some of its bases together with their incident arcs if any. The computational complexity of the problem has been studied in [20], [27], [28]. We face such a problem for arc diagrams without deleting any arcs (*i*, *j*), whose paired nucleotide *j* is inside the considered substructure. Furthermore, we do not impose restrictions for the paired nucleotide *i*. The reason for this choice concerns the nature of the folding process: a nucleotide can perform a hydrogen bond with another already synthesized one. In our formalism, the nucleotide *i* of the pair (*i*, *j*) is synthesized before nucleotide *j*. Operationally, we enumerate the loops in the structure starting from the one whose last nucleotide is the most right, and we extract substructures determined by *M* consecutive loops *L*_*i*_. An illustration of the APS problem and the one with our restriction is given in Figure 6, respectively. In Figure 6B we find only an occurrence of the pattern, graphically identified by bold arcs into the structure. We observe that the pattern occurrences are two and one composed of loops determined by pairs (2, 9) and (6, 11), the other formed by (6, 11) and (10, 16), without considering the restriction.

**Figure 7:**
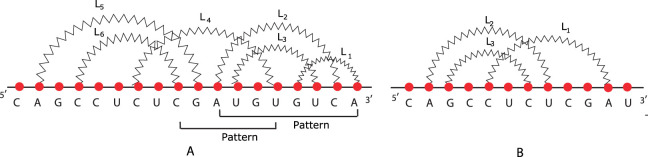
(a) The structure and (b) the pattern.

For each RNA secondary structure represented as an arc diagram, we uniquely determine its relation matrix using Algorithm 3, determination of the relation matrix. The algorithm, whose pseudocode is reported in Appendix A, takes as input the set *B* of the pairs (*i*_*s*_, *j*_*s*_) and returns a matrix, whose element *a*_*k*,*t*_ represents the relation between the loops *L*_*k*_ and *L*_*t*_. It is computed with time complexity of $O\left(N^{2}\right)$ , where *N* is the number of loops in the structure that corresponds to the cardinality of *B*, i.e., the set of pair list of the arc diagram D . As an example, we take into account the RNA secondary structures illustrated in Figure 7. The relation matrices of the two molecules, obtained by the relation matrix algorithm, whose pseudocode is reported in Appendix A, are shown in Table 3. To search a given structural pattern into an RNA secondary structure with arbitrary pseudoknots taking advantage of relation matrices, we define Algorithm 4, structural relation matching. It finds the matrix of the pattern into the matrix of the structure, and it takes as input the two matrices and returns another matrix, whose rows identify the set of loops that forms an occurrence of the pattern in the structure. It is a brute-force search algorithm, whose complexity can be reduced by using techniques of dynamic programming. Continuing with the structures illustrated in Figure 7, we consider the molecule in Figure 7B as a pattern to find into the molecule shown in Figure 7A. The structure contains the pattern twice: the former is determined by loops, *L*_1_, *L*_2_, *L*_3_, while the latter is formed by loops, *L*_4_, *L*_5_, *L*_6_. In general, the output of the structural relation matching algorithm is a matrix characterized by *M* columns and *P* rows, where *M* is the number of loops of the pattern and *P* is the number of occurrences of the pattern in the structure.

**Table 3:** Relation matrices of the structures in Figure 7, respectively. The two patterns into the structure are formed by loops *L*_1_, *L*_2_, *L*_3_ and *L*_4_, *L*_5_, *L*_6_.

	*L*_1_	*L*_2_	*L*_3_	*L*_4_	*L*_5_	*L*_6_
*L*_1_	–	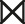	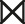	⊙	⊙	⊙
*L*_2_		–	⋒	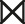	⊙	⊙
*L*_3_			–	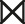	⊙	⊙
*L*_4_				–	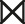	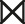
*L*_5_					–	⋒
*L*_6_						–
	*L*_1_	*L*_2_	*L*_3_			
*L*_1_	–	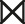	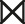			
*L*_2_		–	⋒			
*L*_3_			–			

In the literature, as mentioned in the Introduction, different approaches have been introduced to extract patterns of RNA molecules using several data structures, including arc-annotated sequences, affix trees, and suffix arrays. In Table 4, we report some approaches underlying the data structure used, the performance in terms of computational cost, supporting pseudoknots.

**Table 4:** Comparison of the method (structural matching) with other proposed approaches to extract the RNA substructures.

Approach	Data structure	Computational cost	Pseudoknots	Sequence/structure
Exact pattern matching	Affix tree	O(n⋅m)	No	Both
Exact the largest common substructure	Affix array	O(n⋅m)	No	Both
Maximum arc-preserving common	Arc-annotated sequence	NP	Yes	Both
subsequence				
Arc preserving subsequence problem	Arc-annotated sequence	NP	Yes	Both
The longest arc-annotated	Arc-annotated sequence	NP	Yes	Both
subsequence problem				
Structural matching	Relation matrix	O(n⋅m)	Yes	Structure

*n* and *m* are the length of the pattern and the structure.

Our approach allows us to search a given structural pattern into an RNA secondary structure with arbitrary pseudoknots in *O*(*n* ⋅ *m*) by considering pairwise relations between loops. It corresponds to the arc-preserving subsequence problem with a particular restriction. In particular, we require that no arcs will be deleted if the last paired nucleotide is inside the considered substructure. This constraint follows the folding process: each nucleotide performs a hydrogen bond with another already synthesized one. In our formalism, the first nucleotide of a loop is synthesized before the last one. This restriction allows us to extract patterns, which correspond to the local substructure taking into account the folding process. This approach has a strong impact on the RNA structures analysis because it can consider the structural formations of the molecule.

## 4 Applications

**Figure 8:**
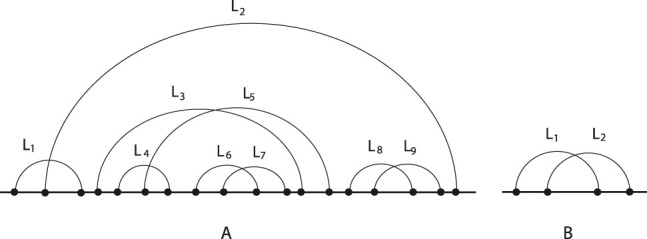
(A) The shadow of molecules with ID PDB_00478, PDB_00408, PDB_00436, PDB_00338, PDB_00589 from RNA strand database; (B) a pattern.

We test our approach on a set of 16S ribosomal RNAs of *T. thermophilus*. The aim is to study the effect of some inhibitors, including antibiotics, tetracycline, hygromycin B. Accordingly, we take into account the RNA molecules with ID PDB_00478, PDB_00408, PDB_00436, PDB_00438, PDB_00589 from the RNA strand database [26]. RNA Strand is a database containing known secondary structures of any type and organism drawn from public databases, searchable and downloadable in several formats. It is an easy online tool for searching, analyzing, and downloading user-selected entries, and is publicly available at http://www.rnasoft.ca/strand. In our experiment, we first consider the shadows of the selected molecules (that we compute applying the algorithm shadow determination). We observe that the same shadow, shown in Figure 8A, characterizes each of the molecules. Therefore, the shadow is not able to capture the effects of the inhibitors over these molecules by confirming that the inhibitors act locally. The antibiotics bind to discrete sites on the 16S submit to effect on ribosome function [29]. To check our algorithm, we compute the relation matrix, and we trivially observe that the pattern, illustrated in Figure 8B, is contained in each shape twice.

**Figure 9:**
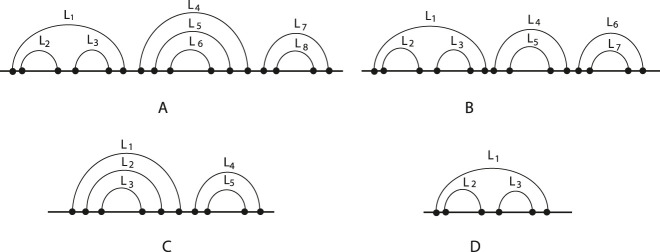
(A) The core of the substructure (from 800th to 900th nucleotides) of molecules with ID PDB_00478, PDB_00408, PDB_00436, PDB_00338 from RNA strand database; (B) the core of the substructure (from 800th to 900th nucleotides) of molecules ID PDB_00589; (C) and (D) two patterns.

Moreover, we consider the core of these molecules between the 800th and 900th nucleotides. The core of substructure molecules with ID PDB_00478, PDB_00408, PDB_00436, PDB_00438 is shown in Figure 9A, while the core illustration of substructure PDB_00589 is in Figure 9B. Finally, the patterns that we identify in the cores are shown in Figure 9C and D. In this case, we observe that the one in Figure 9C is not present in the molecules PDB_00589, while it involves loops *L*_4_, *L*_5_, *L*_6_, *L*_7_, *L*_8_ of the other substructures. However, the pattern in Figure 9D occurs once involving loops *L*_1_, *L*_2_, *L*_3_. In Appendix B, we report the relation matrices and the pattern occurrences in RNA structures of the considered molecules. This result shows that the inhibitors do not influence the molecules between 800th to 900th nucleotides. However, we are able to capture and quantifie the structural changes due to the codon and near-cognate tRNA anticodon stem-loop presence (PDB_00589 molecule).

## 5 Conclusion and future works

RNA functions depend on their three-dimensional configuration. Understanding the relationship between structure and biological function has been considered one of the challenges in biology. In this work, we have faced the problem of identifying a given structural pattern into secondary structures of RNA and RNA–RNA interactions or their abstractions (cores and shadows) with arbitrary pseudoknots. We have used algebraic operators to formalize such RNA secondary structures and their abstractions as a combination of loops. Moreover, we have defined procedures to represent the secondary structure in terms of loops to determine the core and shadows. Finally, we have defined two procedures: determination of the relation matrix and structural relation matching. The former maps each RNA secondary structure into a matrix and the latter identifies each pattern of the RNA structure by searching for a submatrix. We have implemented the proposed methodology in Python, and we have tested our approach on a set of 16S ribosomal RNAs of *T. thermophilus* to understand the effects of some inhibitors. The Python code is available on https://github.com/michelaquadrini/RNARelationPattern. The results show that the approach can capture the local, intermediate and global structural changes by extracting patterns from the molecule, its core, and its shadow, respectively, and taking into account the folding of RNA molecules. The approach can be applied in different scenarios with different aims.

Now, we are working on the tool development by improving its computational performance and making it user-friendly for biologists. Moreover, we want to add other molecular encodings as accepted input, i.e., including dot-bracket and CT files. Dot-bracket is a notation used to encode RNA secondary structure topology, and CT is a format that describes molecules and chemical reactions. Both represent the secondary structures of RNAs and RNA–RNAs interactions as well as the Bpseq notation. However, adding all these input types makes the tool more user-friendly by avoiding format change problems due to the non-existence of a universal notation for encoding secondary RNA structures.

Moreover, we are analyzing RNAs of 16S ribosomal of *T. thermophilus* and *Escherichia coli* to evaluate the effects of inhibitors as a function of thermal differences. This evaluation will be carried out in collaboration with experts of the biological domain to test the impact of our approach on biology. In future work, we want to generalize the approach considering the sequences of nucleotides. In other words, we want to face the problem of finding a given structural pattern into an RNA with arbitrary pseudoknots taking into both the primary and secondary structure of molecules. Although functional RNAs exhibit a highly conserved secondary structure with little sequence similarity, the sequence influences the molecular interactions. In other words, the nucleotide sequence plays a role in the study and prediction of the RNA–RNA interaction structures.

## Appendix A

In this appendix, we define the pseudocode of four algorithms mentioned in the paper, loop determination, core determination, determination of the relation matrix, structural relation matching.

**Algorithm 1:** Loop determination.

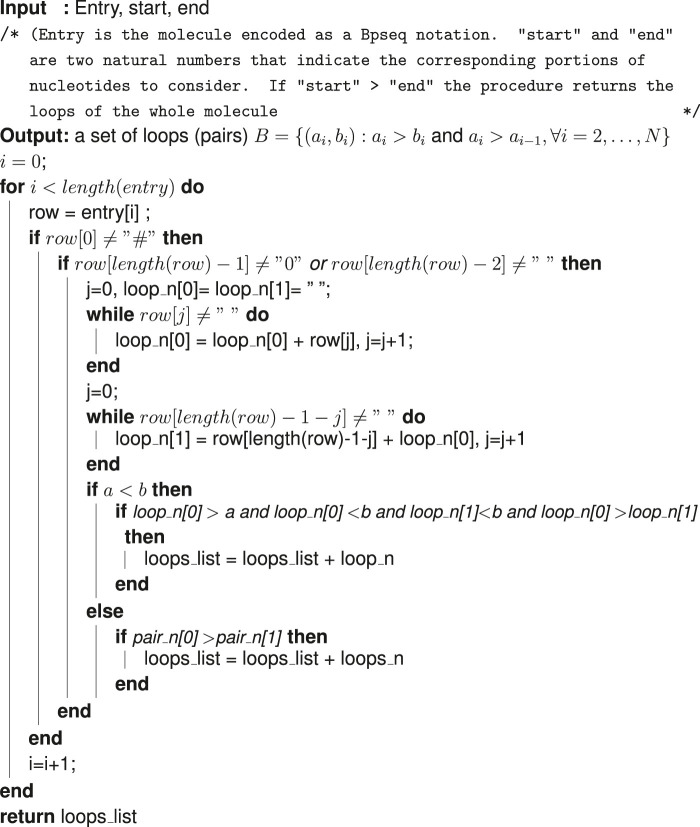

**Algorithm 2:** Core determination.

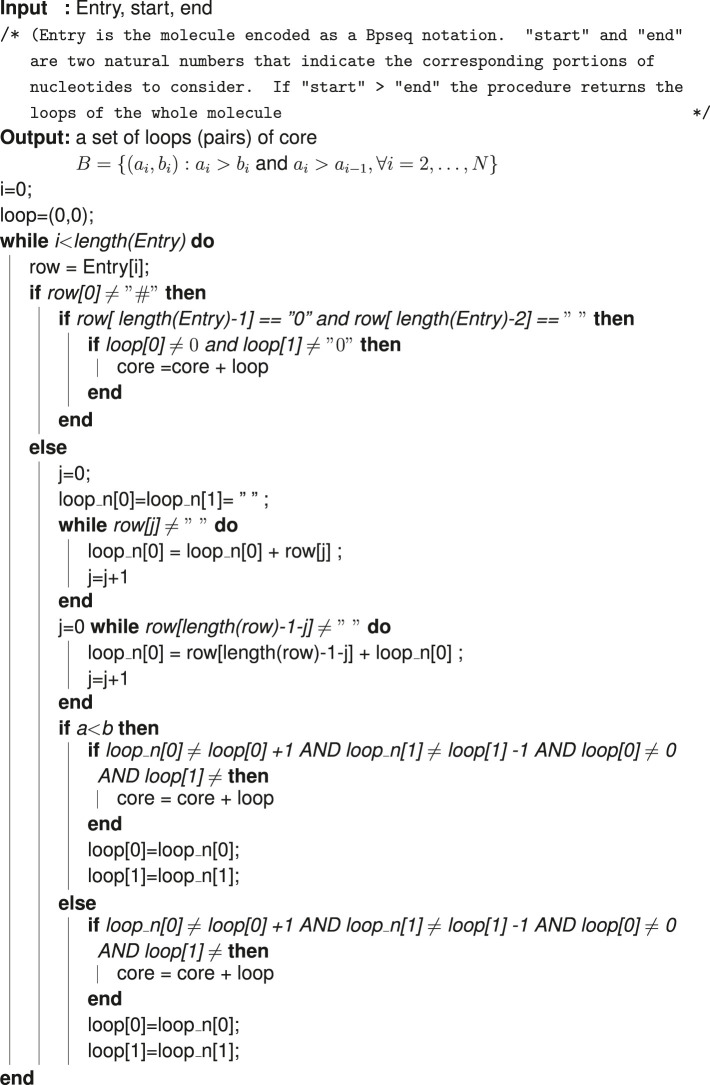

**Algorithm 3:** Determination of the relation matrix.

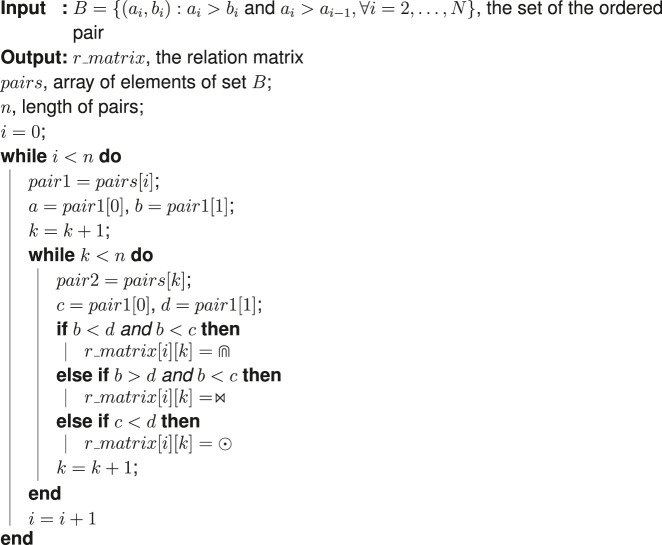

**Algorithm 4:** Structural relation matching.

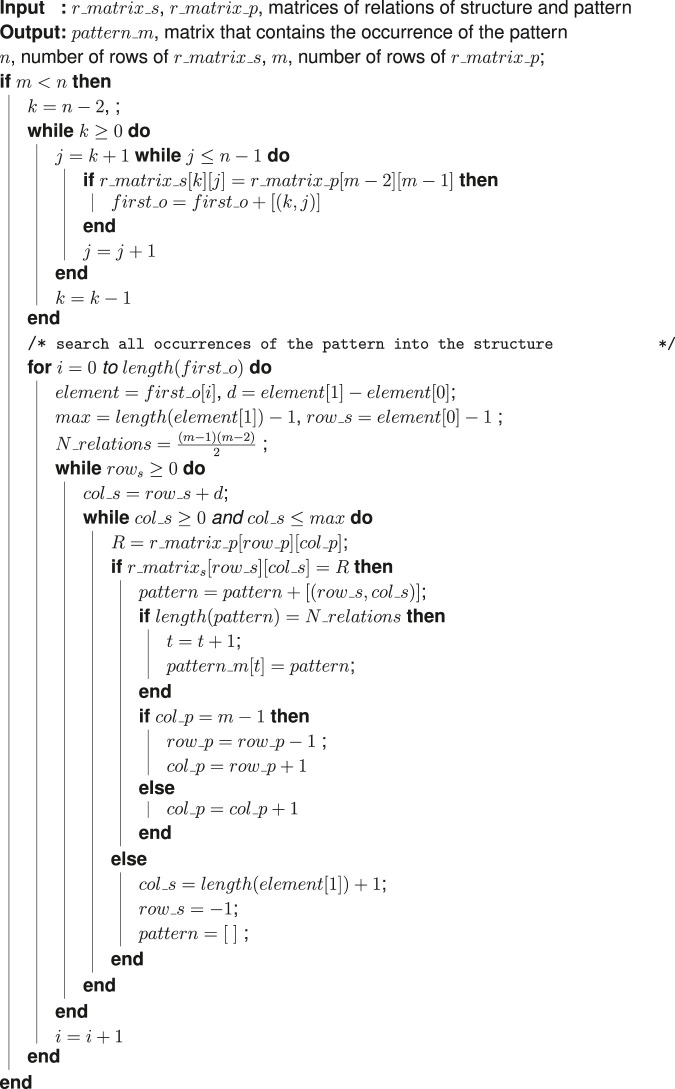

## Appendix B

To test out approach, we selected the molecules with the following ID from RNA strand database [26]

– PDB_00408
– PDB_00436
– PDB_00438
– PDB_00478
– PDB_00589

Each of them is characterized by the same shadow, whose relation matrix is in Table 5.

We extracted substructure from the 800 to 900 nucleotides of the selected molecules and we determined the core. The relation matrix of the core of the substructure (from 800th to 900th nucleotides) PDB_00408, PDB_00436, PDB_00438, PDB_00478 is shown in Table 6, while the relation matrix of core associate to the substructure with ID PDB_00589 is presented in Table 7.

**Table 5:** Relation matrix of the shadow, illustrated in Figure 8, associated to PDB_00408, PDB_00436, PDB_00438, PDB_00478, PDB_00589.

	*L*_1_	*L*_2_	*L*_3_	*L*_4_	*L*_5_	*L*_6_	*L*_7_	*L*_8_	*L*_9_
*L*_1_	–		⊙	⊙	⊙	⊙	⊙	⊙	⊙
*L*_2_		–	⋒	⋒	⋒	⋒	⋒	⋒	⋒
*L*_3_			–	⋒		⋒	⋒	⊙	⊙
*L*_4_				–		⊙	⊙	⊙	⊙
*L*_5_					–	⋒	⋒	⊙	⊙
*L*_6_						–		⊙	⊙
*L*_7_							–	⊙	⊙
*L*_8_								–	
*L*_9_									–

**Table 6:** Relation matrix of the core, illustrated in Figure 9A, of the substructure (from 800th to 900th nucleotides) PDB_00408, PDB_00436, PDB_00438, PDB_00478.

	*L*_1_	*L*_2_	*L*_3_	*L*_4_	*L*_5_	*L*_6_	*L*_7_	*L*_8_
*L*_1_	–	⋒	⋒	⊙	⊙	⊙	⊙	⊙
*L*_2_		–	⊙	⊙	⊙	⊙	⊙	⊙
*L*_3_			–	⊙	⊙	⊙	⊙	⊙
*L*_4_				–	⋒	⋒	⊙	⊙
*L*_5_						⋒	⊙	⊙
*L*_6_						–	⊙	⊙
*L*_7_							–	⋒
*L*_8_								–

**Table 7:** Relation matrix of the core, illustrated in Figure 9B, of the substructure (from 800th to 900th nucleotides) PDB_00589.

	*L*_1_	*L*_2_	*L*_3_	*L*_4_	*L*_5_	*L*_6_	*L*_7_
*L*_1_	–	⋒	⋒	⊙	⊙	⊙	⊙
*L*_2_		–	⊙	⊙	⊙	⊙	⊙
*L*_3_			–	⊙	⊙	⊙	⊙
*L*_4_				–	⋒	⊙	⊙
*L*_5_					–	⊙	⊙
*L*_6_							⋒
*L*_7_							–

The relation matrixes of the two molecules (or patterns), illustrated in Figure 9C and Figure 9D, are shown in Tables 8 and 9.

**Table 8:** Relation matrix of the core, illustrated in Figure 9B, of the substructure (from 800th to 900th nucleotides) PDB_00589.

	*L*_1_	*L*_2_	*L*_3_	*L*_4_	*L*_5_
*L*_1_	–	⋒	⋒	⊙	⊙
*L*_2_		–	⋒	⊙	⊙
*L*_3_			–	⊙	⊙
*L*_4_				–	⋒
*L*_5_					–

**Table 9:** Relation matrix of the core, illustrated in Figure 9B, of the substructure (from 800th to 900th nucleotides) PDB_00589.

	*L*_1_	*L*_2_	*L*_3_
*L*_1_	–	⋒	⋒
*L*_2_		–	⊙
*L*_3_			–

The occurrence of pattern shown in Figure 9C in the structures illustrated in Figure 9A is (*L*_7_; *L*_8_); (*L*_6_; *L*_7_); (*L*_6_; *L*_8_); (*L*_5_; *L*_6_); (*L*_5_; *L*_7_); (*L*_5_; *L*_8_); (*L*_4_; *L*_5_); (*L*_4_; *L*_6_); (*L*_4_; *L*_7_); (*L*_4_; *L*_8_), while there is no occurrence of the patterns for the structures illustrated in Figure 9B. The pattern Figure 9D occurs in all considered structures once. The occurrence is (*L*_2_; *L*_3_); (*L*_1_; *L*_2_); (*L*_1_; *L*_3_).
